# Decline in Vaccination Coverage against Poliomyelitis in the municipalities of Vale do Paraíba (SP) under a spatial approach

**DOI:** 10.1590/1984-0462/2024/42/2023137

**Published:** 2024-07-08

**Authors:** Maria Carolina Ladeira de Carvalho, Paola Carvalho Lioi, Vitoria Nallin de Godoy, Adriana de Oliveira Ribeiro Santos, Luiz Fernando Costa Nascimento

**Affiliations:** a Universidade de Taubaté, Taubaté, SP, Brazil.; b Universidade Estadual Paulista "Júlio de Mesquita Filho", Guaratinguetá, SP, Brazil.

**Keywords:** Vaccination, Poliomyelitis, Vaccine coverage, Child health, Vacinação, Poliomielite, Cobertura vacinal, Saúde da criança

## Abstract

**Objective:**

To analyze vaccination coverage (VC) for polio in the municipalities of Vale do Paraíba in the State of São Paulo.

**Methods:**

This is an ecological and exploratory study of VC in 35 municipalities using a spatial approach; VC data were obtained from the IT Department of the Unified Health System (DATASUS), for the years 2015 and 2019, and categorized into Low (VC<95%) and ideal (≥95%). Information was obtained on gross domestic product (GDP), professional rates and number of basic health units (UBS) and maternal data such as age, marital status (MS) and education. Univariate and bivariate Moran indices were estimated for the years 2015 and 2019, and thematic maps were created for CV values.

**Results:**

The average VC values were 107.7%±27.2 in 2015, and 94.2%±27.8 in 2019 (p<0.05). In 2015 vs. 2019, there were 10 vs. 25 municipalities in the Low category. In 2015, the variables VC, number of UBS, age, education, and MS were spatially correlated, but in 2019 only maternal age and education were spatially correlated. The bivariate Moran was significant and negative for VC in 2019 with maternal education. There was an increase in municipalities with worsening VC values.

**Conclusions:**

The spatial approach identified a decrease in polio vaccination coverage in the studied region.

## RESUMO

**Objetivo:**

Analisar as coberturas vacinais (CV) da poliomielite nos municípios do Vale do Paraíba paulista.

**Métodos:**

Trata-se de um estudo ecológico e exploratório de CV em 35 municípios com abordagem espacial. Os dados de CV foram obtidos do Departamento de Informática do Sistema Único de Saúde (DATASUS), referentes aos anos de 2015 e 2019 e categorizados em baixa (CV<95%) e ideal (≥95%). Foram obtidas informações sobre Produto Interno Bruto (PIB), taxas de profissionais, número de unidades básicas de saúde (UBS) e dados maternos como idade, estado civil (EC) e escolaridade. Foram estimados índices de Moran univariados e bivariados para os anos de 2015 e 2019 e criados mapas temáticos para valores de CV.

**Resultados:**

Os valores médios de CV foram de 107,7%±27,2 em 2015 e 94,2%±27,8 em 2019 (p<0,05). Em 2015, eram dez municípios na categoria baixa e, em 2019, eram 25 municípios. Em 2015, as variáveis CV, número de UBS, idade, escolaridade e situação marital foram correlacionadas espacialmente, mas em 2019 apenas idade materna e escolaridade foram correlacionadas espacialmente. O Moran bivariado foi significativo e negativo para CV em 2019 com escolaridade materna. Houve aumento de municípios com piora nos valores de CV.

**Conclusões:**

A abordagem espacial identificou diminuição da cobertura vacinal contra a poliomielite na região estudada.

## INTRODUCTION

Vaccination is considered one of the most successful public health strategies.^1,2^ The application of immunizers in early childhood is of great importance in the full development and reduction of infant mortality rates, protecting against serious infectious diseases, some of which are highly transmissible and others disabling.

The National Immunization Program (PNI) of the Brazilian Ministry of Health, established in 1973, is one of the most complete immunization programs in the world, and has records of great achievements with regard to the control, reduction and/or elimination of vaccine-preventable diseases. Following the eradication of smallpox, the 1^st^ National Vaccination Campaign against Poliomyelitis began in 1980, with the goal of vaccinating all children under 5 years of age in a single day. The last case of poliomyelitis in Brazil occurred in Paraíba in March 1989. In 1994, Brazil, together with the other countries in the Americas, received from the International Commission the Certification of Absence of Autochthonous Circulation of Wild Poliovirus in the Americas.^3^

Nonetheless, a nationwide reduction in vaccination coverage (VC) rates has been identified in recent years, and this reduction signals a problem for collective immunity and the risk of resurgence of diseases hitherto controlled or eradicated.^4^ Several factors may be associated to the drop in VC, such as socioeconomic and demographic factors, low maternal education, worse living conditions, size of the family nucleus, outpatient care, shortage of vaccines, fake news, lack of risk perception for diseases, access to the vaccination service, among others.^5–12^

Locations, whether municipalities or microregions, with low vaccination coverage can be hidden by calculating the general VC indicator in relation to the estimated population residing in the state or country. In this sense, studies are needed to assess VC in municipalities, with the aim of identifying the situation in specific regions and directing strategies and health policies. A way of approaching this theme can be the spatial analysis, which can indicate the presence of clusters of municipalities, in the case of this study, but being used in national studies to investigate injuries and diseases,^13–16^ and recently in the coverage vaccination.^17–18^

The objective of this study is to analyze the vaccination coverage for poliomyelitis in the municipalities of Vale do Paraíba between 2015 and 2019 using spatial analysis tools.

## METHOD

**Figure 1:**
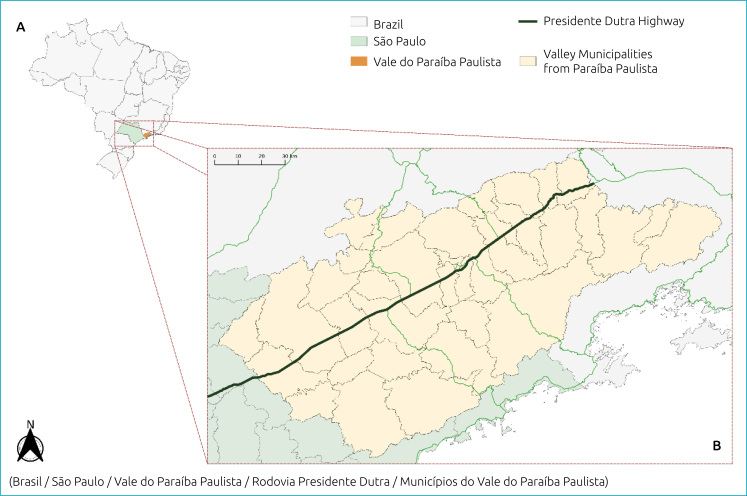
Location of Vale do Paraíba Paulista (B) in the State of São Paulo and Brazil (A).

An ecological and exploratory study of vaccination coverage (VC) was carried out in 35 municipalities, that which formed the basis for the spatial analysis, in the Vale do Paraíba, region State of São Paulo, using a spatial approach. The region of Vale do Paraíba is located between the two largest Brazilian cities (São Paulo and Rio de Janeiro). The population is approximately 2.2 million inhabitants (http://tabnet.datasus.gov.br/cgi/tabcgi.exe?ibge/cnv/poptsp.def – access 21/nov/2023), and the region is crossed by the Presidente Dutra Highway, the busiest in Brazil, and by Carvalho Pinto and Tamoios Highways (Figure 1). It is bordered to the north by the Mantiqueira Mountains and to the south by another mountain chain, Serra do Mar. It is an important industrial center, with universities such Universidade de São Paulo, Universidade do Estado de São Paulo, Universidade de Taubaté, and research institutes such as the National Institute for Space Research and the Technological Institute of Aeronautics.

Data regarding VC for polio applied in the first year of life were obtained from the DATASUS website (https://datasus.saude.gov.br/acesso-a-informacao/imunizacoes-desde-1994/accessed on 12/02/2023), with values shown in percentages, as well as the doses applied with this immunizer in the years 2015 and 2019. This coefficient is calculated as a ratio between the applied doses of a given vaccine and live births in a given city in a given year, in percentage. The coefficient aims to identify possible falls, because there may be a compromise in the protection of the child and also of the community as a whole.

Information was obtained on the socioeconomic index of these municipalities, such as the Gross Domestic Product (GDP), in Reais, for the year 2013, the latest data (http://tabnet.datasus.gov.br/cgi/deftohtm.exe?ibge/cnv/pibmunbsp – access 04/03/2023). Also, for each municipality, information was gathered on the DATASUS website about the number of professionals who are part of the family health strategy, such as the family health doctor, the pediatrician and the family health nurse (proportions according to the populations of each municipality based on 100,000 inhabitants), and on the number of basic health units in each municipality (proportions according to the populations of each municipality based on 1000 inhabitants). Maternal variables selected from the years 2015 and 2019 were maternal age (those aged between 10 and 19 years and over 35 years), maternal education (8 years and more of schooling) and marital status (those living without a partner, i.e, single, widowed, divorced). These variables were analyzed as a proportion in relation to live births in the respective years.

The digital grid of the municipalities in this study was obtained from the Brazilian Institute of Geography and Statistics for the year 2015, for the State of São Paulo (https://www.ibge.gov.br/geociencias/organizacao-do-territorio/malhas-territorialis/15774-malhas.html?=&t=downloads – accessed on 12/02/2023). An information bank was created in the TerraView software (National Institute for Space Research-INPE; https://www.dpi.inpe.br/).

Thematic maps were created with VC data for the years 2015 and 2019, and were stratified in two categories: VC<95% (Low), and VC≥95% (Ideal). A thematic map was constructed for the difference between the percentages in 2019 and 2015 to identify possible differences between the two periods, and it was categorized as Worse, when the difference was negative (VC in 2019 was lower than the VC in 2015), and Better, when the difference was positive.

Univariate global Moran index (M_I_) values were obtained for these attributes. This index varies between [-1 and +1]; positive values mean that the municipalities have similarities according to a certain attribute (high rates, for example), with possible visual identification of the occurrence of clusters; negative values indicate that there are municipalities with high rates surrounded by municipalities with low rates. For the study of two spatially georeferenced variables, Moran's bivariate index, denoted as I_xy_, is an index of spatial correlation between the study variable (dependent) and the independent variables obtained in 35 municipalities.^19^

The bivariate Moran index was used in the case of this study vaccination coverage a dependent variable, and the independent variables were maternal age below 20 years old and above 35 years old, marital status of those who live without a partner (widow, single or divorced), and maternal education of those with more than 8 years of schooling, as well as care and access to health, where the bivariate Moran index was used. The classes of these variables were described above. For this analysis, the public access software GeoDa, available from the University of Chicago (https://spatial.uchicago.edu), was used.

The student's t test was used to compare means when indicated. The significance level adopted in this study was alpha=5%. This study was not submitted to the Research Ethics Committee because it used publicly available data and without the possibility of identifying the subjects.

## RESULTS

The mean values and respective standard deviations (sd) of vaccination coverage were 107.7%±27.2 in the year 2015, and 94.2%±27.8 in the year 2019; these percentages of vaccination coverage were statistically different (p<0.05). There was a decrease in doses applied between 2015 – 28,831 doses and 2019 – 25,941 doses.

**Figure 2:**
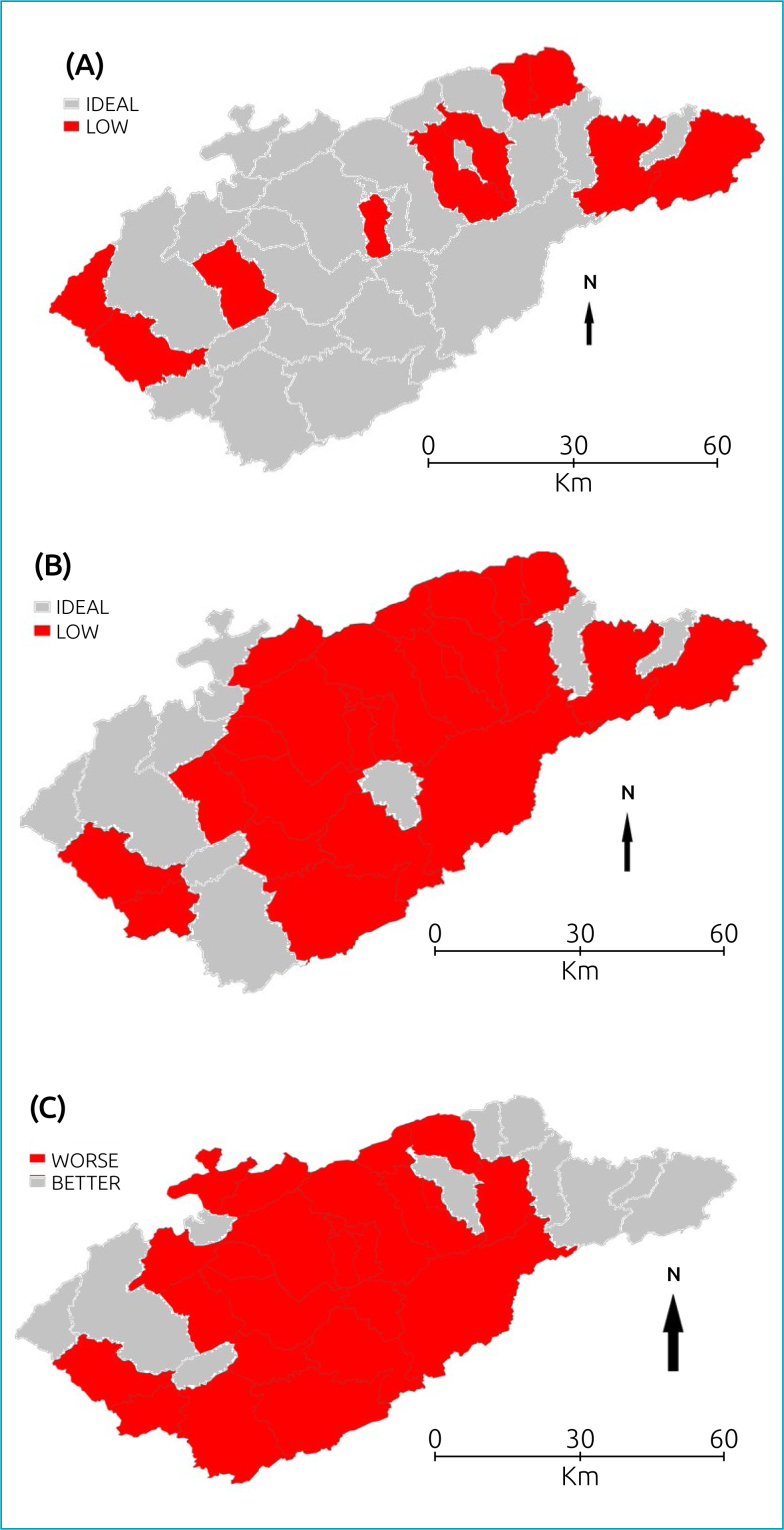
Thematic maps for Vaccination Coverage (VC) in 2015 (1-A), in 2019 (1-B), and differences (1-C) between VC in 2019 and 2015, Vale do Paraiba, SP, 2015–2019.

In 2015, ten municipalities were in the Low category and 25 (71.4%) in the ideal category, these latter being located along the axis of Via Dutra and those located in the Low category outside this road axis, both towards Serra do Mar and towards Serra da Mantiqueira (Figure 2A). In 2019, there was a decrease in vaccination coverage, with 25 municipalities being in the Low, and 10 municipalities (28.6%) in the Ideal category. (Figure 2B)

The proportions of health professionals (PROF) were different for the years of study, increasing in 2019 (p<0.05). There was no significant change in the proportions of basic health units (UBS). The mean, minimum and maximum values, and the respective standard deviations and p-values of the study variables are shown in Table 1.

**Table 1:** Mean, minimum and maximum (Min-Max) values, in percentages, and respective standard deviations, and p-values of the study variables, Vale do Paraíba, 2015 and 2019.

	Mean (sd)	Min-Max	p-value*
VC 2015 (%)	107.7 (27.2)	62.0–177.1	<0.05
VC 2019 (%)	94.2 (27.8)	21.0–185.7	
PROF–2015	10.0 (2.9)	4.9–18.2	<0.05
PROF–2019	12.3 (3.6)	5.4–19.4	
UBS–2015	0.33 (0.21)	0.06–1.03	NS
UBS–2019	0.36 (0.23)	0.06–1.03	
MATER EDU–2015	78.3 (10.0)	48.4–100.0	<0.05
MATER EDU–2019	90.7 (5.4)	70.6–97.7	
MARI STAT–2015	45.6 (9.6)	23.5–66.7	<0.05
MARI STAT–2019	54.5 (12.6)	33.3–91.7	
MATER AGE–2015	30.9 (3.4)	23.9–39.5	NS
MATER AGE–2019	31.1 (6.3)	22.7–58.3	

* provided by Student's *t*-test. VC 2015: Vaccination coverage (VC) in 2015; VC 2019: Vaccination coverage in 2019; PROF–2015: proportion of health professionals per 1000 inhabitants in 2015; PROF–2019: proportion of health professionals per 1000 inhabitants in 2019; UBS–2015: proportion of Basic Health Units per 1000 inhabitants in 2015; UBS–2019: proportion of Basic Health Units per 1000 inhabitants in 2019; MATER EDU: maternal education; MARI STAT: marital status; MATER AGE: maternal age; NS: not significant (p>0.05).

The values of the univariate Moran indices for each municipality are shown in Table 2. There are significant similarities between the municipalities in terms of maternal age in 2015 – smaller proportions of mothers aged less than 20 years and over 34 years are located outside the Via Dutra axis and in the extreme east; as well as for maternal education, both in 2015 and in 2019, the highest proportions being in municipalities along Via Dutra. Municipalities with more than 50% of mothers living without a partner are located in the middle area of Vale do Paraíba, towards the extreme east in 2015, and in 2019 the distribution is very similar. These maps are not shown.

**Table 2:** Univariate Moran index (I_M_) values and respective p-values of the study variables, for the years 2015 and 2019, Vale do Paraíba, 2015, 2019.

	I_M_(p-value)
VC 2015	-0.20 (0.02)
VC 2019	-0.02 (0.49)
PROF 2015	0.03 (0.37)
PROF 2019	-0.10 (0.21)
UBS 2015	0.19 (0.02)
UBS 2019	0.17 (0.08)
MATER EDU–2015	0.23 (0.03)
MATER EDU –2019	0.17 (0.04)
MARI STAT–2015	0.36 (0.01)
MARI STAT–2019	0.15 (0.12)
MATER AGE–2015	0.28 (0.04)
MATER AGE–2019	0.26 (0.04)
GDP	0.16 (0.11)

VC 2015: Vaccination coverage (VC) in 2015; VC 2019: Vaccination coverage in 2019; PROF 2015: proportion of health professionals per 1000 inhabitants in 2015; PROF 2019: proportion of health professionals per 1000 inhabitants in 2019; UBS 2015: proportion of Basic Health Units per 1000 inhabitants in 2015; UBS 2019: proportion of Basic Health Units per 1000 inhabitants in 2019; MATER EDU: maternal education; MARI STAT: marital status, MATER AGE: maternal age; GDP: Gross Domestic Product.

For vaccination coverage rates, in 2015, there were municipalities with high VC surrounded by municipalities with low VC; this fact was pointed out by the I_M_ value with a negative value, although significant; in 2019 this distribution of VC in the municipalities was similar, but not statistically significant. Comparing the thematic maps of VC distributions in 2015 (Figure 2A) and 2019 (Figure 2B), there is an increase in municipalities with worsening VC values. In 2015 there were 10 municipalities with VC of less than 95% (22.9%) and in 2019 there were 25 (71.4%); comparing data from 2015 and 2019, there was an increase in vaccination coverage in 11 municipalities (Figure 2C).

As for the results of the bivariate Moran index (Table 3), GDP was not significantly associated with VC, but with opposite results; in 2015, the higher the GDP value, the better the vaccination coverage, which is consistent; in 2019, the opposite occurred, that is, coverage was lower in municipalities with better GDPs. Attention was drawn to the fact that the VC was lower when the independent variable was maternal education equal to or greater than 8 years of schooling, being negative and significant in the year 2015; in 2019, the role of mothers with maternal education equal to or greater than 8 years of schooling had a negative impact, but without statistical significance. Mothers who lived without a partner were positively correlated with better vaccination coverage in both years, also without statistical significance. There was no significant difference for the correlation between maternal age and VC in both periods.

**Table 3:** Bivariate Moran index values for the years 2015–2019, and respective p-values (in parentheses) of the variables corresponding to the years, according to the vaccination coverage in 2015 (VC 2015), and 2019 (VC 2019), Vale do Paraíba, 2015, 2019.

	VC 2015	VC 2019
GDP	0.08 (0.17)	-0.06 (0.20)
PROF	-0.11 (0.08)	-0.03 (0.46)
UBS	-0.04 (0.39)	0.06 (0.17)
MATER EDU	-0.20 (<0.01)	-0.10 (0.11)
MARI STAT	0.07 (0.22)	0.02 (0.49)
MATER AGE	-0.03 (0.32)	0.03 (0.34)

GDP: Gross Domestic Product; PROF: proportion of health professionals per 1000 inhabitants for the years 2015 and 2019; UBS: proportion of Basic Health Units per 1000 inhabitants for the years 2015 and 2019; MATER EDU: maternal education; MARI STAT: marital status; MATER AGE: maternal age.

## DISCUSSION

To the best of our knowledge, this is the first study carried out in the State of São Paulo with data on vaccination coverage against poliomyelitis in the first year of life. This study identified a cluster of municipalities with a high drop in the polio VC rate in the period studied, namely Guaratinguetá, Pindamonhangaba, Tremembé, Taubaté, Potim, Redenção da Serra, Lagoinha, São Luiz do Paraitinga and Natividade da Serra. The difference between the vaccination period coverage was significant. Our findings in Vale do Paraíba show that 25 (71.4%) of the 35 municipalities in the study had VC above 95% in 2015; but only 10 (28.6% of the total municipalities) had the same VC in 2019.

The World Health Organization recommends that immunization programs regularly identify whether there are pockets of groups with low vaccination coverage in the country and, if they exist, investigate the associated factors, this monitoring being the strategic axis of good practices in the management of immunization programs.^20^

Cunha et al. studied the VC of vaccines given to children under 1 year of age in 2016 and 2017 in 223 municipalities in the State of Paraíba, Northeast Brazil, using information from the Live Birth Information System (SINASC) and the Information System of the National Immunization Program (SI-PNI), as well as spatial analysis tools, such as our study, and identified a considerable number of municipalities with low vaccination coverage, particularly against Bacillus Calmette and Guérin (BCG).^18^ In the case of the polio vaccine, in 2016, the coverage considered adequate (>95%) was just under 50% of the municipalities in Paraíba, and in 2017 the VC found was 38%. Higher coverage was found in municipalities in the region of the State of Paraiba called Sertão Paraibano, but they do not indicate possible associated factors.^18^

A study using the spatial analysis of vaccination coverage in children under 1 year of age in the State of Minas Gerais between 2015 and 2020, according to the 19 Regional Health Territories, showed that, in 2020, there was a lower proportion of recommended vaccination coverage for BCG, vaccines with human rotavirus, against pneumococcus 10, pentavalent, against meningococcus C, against yellow fever and against poliomyelitis, and especially with the pentavalent vaccine. The reasons that could be contributing to this coverage may include regional differences associated with structural inadequacies of the vaccination rooms, compromising the availability of vaccines.^17^

A systematic e review of publications from 1992 to 2014 analyzed factors related to childhood immunization of children aged 0 to 24 months. The most cited factors for incomplete or delayed vaccination are the birth order of children followed by low maternal education and low socioeconomic levels. In countries with low to medium GDP, birth outside the health service, forgetting the appointment date and mothers who work outside the home were also cited as causes of incomplete or delayed vaccination.^21^ In the study in Vale do Paraíba, the order of birth was not analyzed. As for the percentage distribution of mothers with 8 years or more of schooling, there was a significant increase between 2015 and 2019; however, this variable was negatively associated with a higher VC when analyzed by the bivariate Moran, that is, municipalities that had a high proportion of mothers with secondary or higher education had their children with lower VC both in 2015 and in 2019, a paradoxical finding for which we still do not have an explanation. On the other hand, these data partially coincide with those of Barata et al., in a study where these authors included vaccines other than the one against poliomyelitis.^5^ A similar situation is pointed out in a review by Hu et al.^8^ and Tauil et al.^21^

Maternal age was another variable studied, with percentages of mothers under 20 years of age and over 34 years of age not being statistically different in 2015 and 2019 (calculated using the Student *t* test). Nevertheless, the Moran index was significant, and the thematic maps of these distributions indicate that most municipalities with higher rates are outside the Via Dutra axis. The role of maternal age in vaccination coverage was not significant when analyzed using the bivariate Moran index result, both in 2015 and in 2019. Similar data were found in a study carried out in the State of Maranhão with an incomplete basic scheme.^22^

Many factors may be acting synergistically for this historical drop in vaccination coverage that has been observed, such as the precariousness of the Unified Health System (SUS), the implementation of the new Information System of the National Immunization Program (SI-PNI); social and cultural aspects that affect acceptance of vaccination; anti-vaccination movements, structural inadequacies of vaccination rooms and inconstancy in the availability of immunobiological agents in Primary Health Care (PHC) services.^21–23^ Due to the extensive national territory, PCH services can present different realities with regional inequalities that may influence and/or contribute to the drop in vaccination coverage in different regions or specific populations.^22^ Studies demonstrate the great importance of PCH professionals, whether during monthly visits to the Health unit or home visits, in detecting vaccine delay.^11^

In the study presented herein, the proportions of health professionals (PROF) and basic health units (UBS) in the studied area increased in both periods, and the spatial distribution of UBS was spatially autocorrelated with the highest proportions of VC in the far east and in municipalities close to Serra do Mar; along Via Dutra these proportions were lower in the municipalities. Using the bivariate Moran index that correlates vaccination coverage (dependent variable) with the variable proportions of health professionals, paradoxically there was a negative autocorrelation, that is, municipalities with higher proportions of health professionals had lower vaccination coverage; it is possible that there are other factors not analyzed in this project, such as birth order or full-time working mother, that could interact with or confound these negative findings. As for the proportion of basic health units (UBS), in 2019, the value of Moran's bivariate index was positive, possibly due to the slight increase in the proportion of UBS offering more places available for vaccination.

The SI-PNI represents a breakthrough in VC data management in Brazil. The registration of vaccine doses in the system is performed nominally, per vaccinated individual, favoring the identification and search of missing individuals, scheduling of doses, and monitoring of VC by neighborhood, basic unit or health region. It calculates the vaccination coverage, by basic unit, municipality, regional unit of the State Department of Health, state, and country. It provides information about the routine and campaigns, abandonment rate and sending of immunization bulletins, as well as inventory management, distribution, and adverse reactions. It is these data that are made available on the DATASUS, and that may have limitations depending on the access and insertion of data in the computerized system.^24–26^ Despite the importance of the SI-PNI, some factors can hinder the generation of information, such as the absence of computers connected to the internet in the vaccination rooms, lack of stable internet and slowness in the system.^17^ Potential information inconsistencies may occur, since the insertion of vaccine doses in the system is performed by several professionals.

The causes for some regions to present VC above 100% may be related to the situation of newborns registered in locations other than where the mother resides, mainly in small towns and without maternity hospitals, as well as the use of vaccination rooms by some users in neighboring municipalities, increasing the number of vaccinees beyond the target population of the respective municipality.

This study shows a decrease in VC in 2019 compared to 2015, even with an increase in the number of health professionals, and some possible explanations may be structural inadequacies in vaccine rooms, unavailability of immunobiological agents and deficiency in the permanent education of health professionals as reported by some authors,^17^ and possibly appointment times that may not meet the families’ demands. Keeping health facilities open full time and extended hours to facilitate access for families could likely improve VC rates.

Studies carried out in different parts of the country showed differences in coverage for different social groups, with lower coverage in the strata of vulnerable children, such as low family income, low educational level of guardians, and high number of children.^5,9^ This is different from our findings in the years 2015 and 2019 regarding maternal schooling of 8 years and more. Probably some of the other factors already mentioned, such as care conditions and adequate access to the SI-PNI system, may be involved.^27^

Although many countries have eliminated polio, it remains endemic in countries such as Afghanistan, Pakistan and Nigeria. As long as there are people infected with polio, other children, in different countries, are at risk of contracting the disease. Joint actions to publicize the importance of vaccination in the field of health and education, actively seeking out children who are behind on vaccines and strengthening the link between health services and the children's families are pillars of the strategy to improve coverage rates against poliomyelitis and other illnesses.^28^

This study has some limitations. The source of data, even being official, may contain inaccuracies, for instance, on information about the number of administered vaccines obtained using the internet or filling out spreadsheets. Also, communication problems can cause errors and delay in the consolidation of data. Another limitation is the possibility of children being vaccinated in other municipalities and those who are vaccinated in private clinics and during campaigns, which could lead to underreporting with a false compromise in vaccination coverage in their municipality of residence.

Despite the limitations, it was possible to show an important decrease in vaccination coverage for poliomyelitis in the region studied and to identify the municipalities with this decrease, providing subsidies for public health strategies.

## Data Availability

The database that originated the article is available with the corresponding author.
